# Brodie’s Abscess in a Pediatric Patient: A Diagnostic Pitfall

**DOI:** 10.7759/cureus.107797

**Published:** 2026-04-27

**Authors:** Ioannis Christofides, Rob Knoef, Esther M Bloemheuvel

**Affiliations:** 1 Orthopaedics and Trauma, Isala Hospital, Zwolle, NLD

**Keywords:** brodie‘s abscess, diagnostic pitfall, pediatric patients, persistent knee pain, subacute osteomyelitis

## Abstract

Brodie’s abscess is a localized form of subacute osteomyelitis that often presents without systemic illness and may be misdiagnosed due to its indolent course. We describe a pediatric patient in whom Brodie’s abscess initially mimicked a less severe, superficial condition, resulting in delayed definitive diagnosis. The patient was initially treated for presumed septic infrapatellar bursitis, with transient clinical improvement under antibiotic therapy before imaging revealed proximal tibial osteomyelitis consistent with a Brodie’s abscess. This case highlights the risk of misdiagnosis in children presenting with atraumatic knee pain and underscores the importance of reconsidering initial diagnoses when clinical progression is atypical. Early and appropriate imaging is essential to avoid delayed diagnosis and potential complications.

## Introduction

Subacute osteomyelitis, commonly referred to as Brodie’s abscess, is a localized form of bone infection characterized by a well-defined intraosseous abscess cavity surrounded by granulation tissue and reactive sclerotic bone. It most frequently affects children and adolescents and typically involves the metaphysis of long bones, particularly the tibia, although other sites such as the femoral neck have also been described [1,2]. In some cases, the lesion may extend across the growth plate into the epiphysis, particularly in skeletally immature patients. The abscess cavity may vary in size and typically contains purulent or mucoid material, while microbiological examination does not always identify a causative organism [1-3].

Unlike acute osteomyelitis, Brodie’s abscess often presents with an indolent clinical course and limited systemic inflammatory response. Patients commonly report localized pain, sometimes with mild swelling, whereas fever and significant systemic illness are frequently absent [1,3,4]. This atypical presentation contributes to diagnostic delay, with symptoms often persisting for weeks or even months before a definitive diagnosis is established [1,3-5].

The diagnosis can be further complicated by its variable clinical and radiological presentation. Early radiographs may be subtle or nonspecific, and Brodie’s abscess can mimic a range of other conditions, including osteoid osteoma, chronic non-bacterial osteomyelitis, and primary bone tumors [1]. As highlighted in recent case reports, this overlap in presentation may lead to misinterpretation and delayed diagnosis, particularly in pediatric patients [1,3,4,6-8].

Although magnetic resonance imaging (MRI) is the modality of choice for evaluating suspected osteomyelitis, the initial clinical presentation may direct attention toward more superficial or less severe pathology, potentially delaying appropriate imaging [1,7,9]. Awareness of this entity and its atypical presentation is therefore essential in children presenting with persistent or unexplained bone pain.

We present a pediatric case of Brodie’s abscess with atypical clinical presentations, highlighting the risk of misdiagnosis and emphasizing the importance of appropriate imaging in children with persistent or recurrent knee pain.

## Case presentation

A 13-year-old boy presented with progressive left knee pain and swelling without preceding trauma. He reported a single episode of subjective fever but was afebrile and clinically stable at presentation.

Physical examination revealed localized anterior knee swelling and warmth without joint effusion. Range of motion was limited due to pain, with flexion to 130° and an extension deficit of 20°. Laboratory investigations demonstrated an elevated C-reactive protein (CRP) of 115 mg/L (reference range: <1 mg/L) with a normal leukocyte count.

Ultrasound revealed an anechoic fluid collection in the infrapatellar bursa without evidence of joint effusion. Based on these findings, a working diagnosis of septic bursitis was made by the pediatrician. The patient was subsequently admitted to the pediatric department, and intravenous flucloxacillin was initiated.

The patient initially showed clinical and biochemical improvement, with a reduction in pain, restoration of range of motion, and a decrease in CRP levels, and was discharged on oral flucloxacillin, completing a total antibiotic course of 14 days. However, six weeks later, he returned with recurrent pain, nocturnal symptoms, and elevated inflammatory markers.

**Figure 1:**
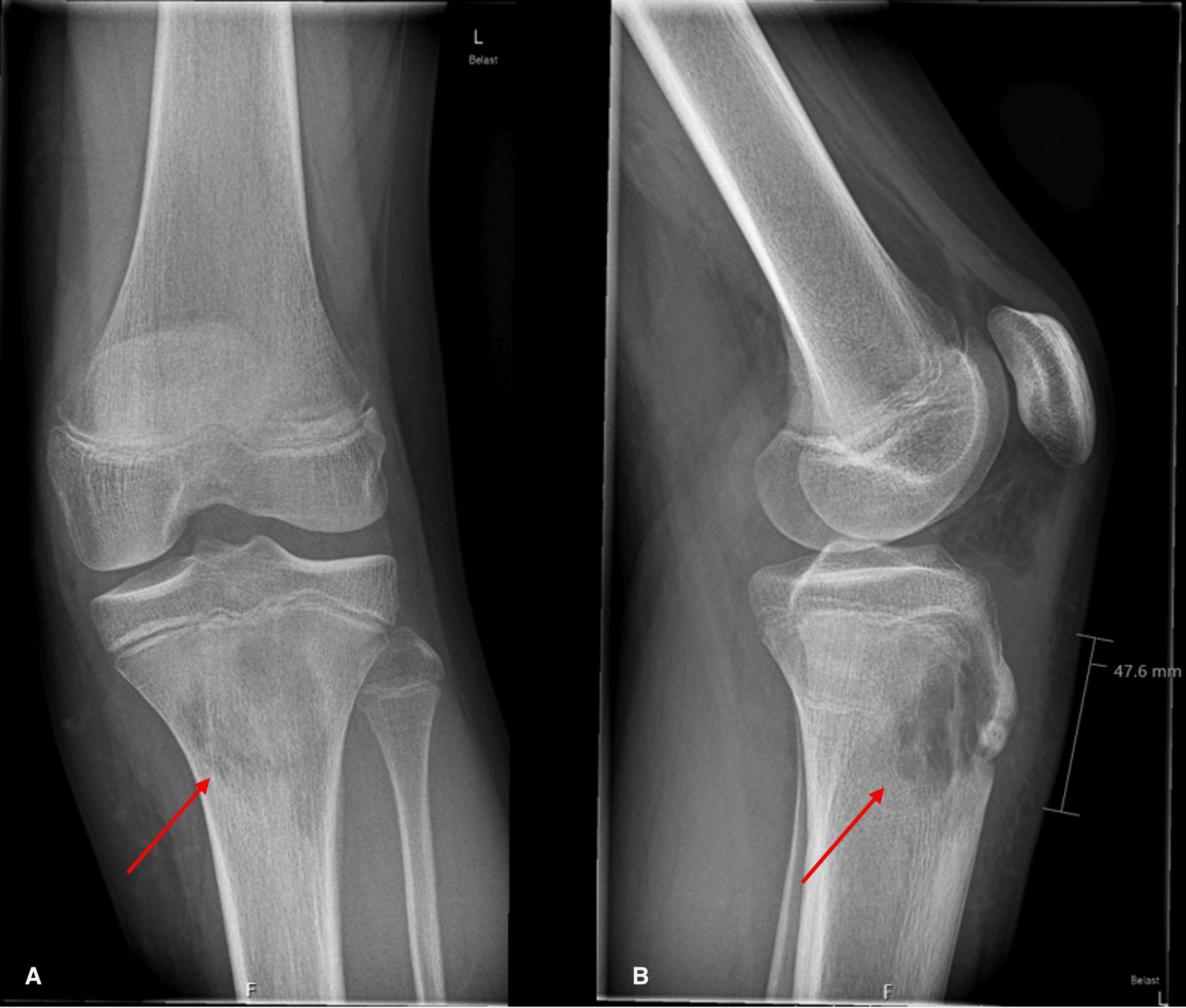
Plain radiograph Plain radiograph of the left knee demonstrating a lytic lesion (red arrow) in the proximal tibial metaphysis with ill-defined margins, suspicious for underlying bone pathology.

**Figure 2:**
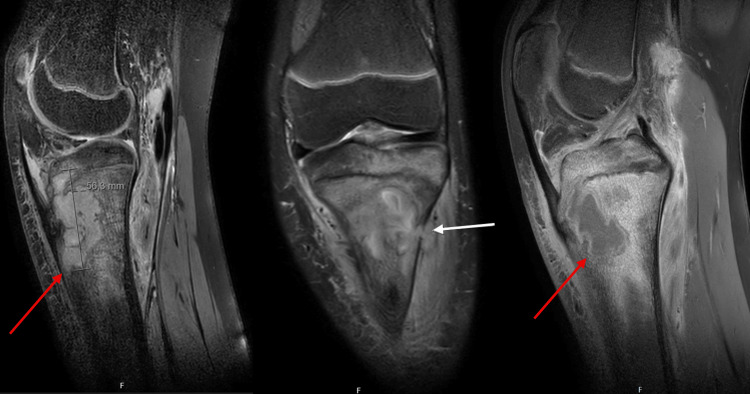
MRI MRI of the left knee showing a large intraosseous abscess cavity in the proximal tibia measuring approximately 56 mm (red arrow), with surrounding bone marrow edema and cortical breach (white arrow). The imaging features are consistent with subacute osteomyelitis (Brodie’s abscess). The T1-weighted image demonstrates a central hypointense cavity surrounded by a hyperintense granulation rim (“penumbra sign”) with adjacent marrow edema.

Plain radiography demonstrated a lytic lesion in the proximal tibia (Figure 1). MRI revealed extensive proximal tibial osteomyelitis with a 56 mm Brodie’s abscess, cortical disruption, and surrounding soft tissue inflammation (Figure 2).

Surgical drainage and debridement of the abscess were performed. The patient was initially started on intravenous cefazolin. Cultures subsequently yielded Streptococcus intermedius. Based on the culture and sensitivity results, antibiotic therapy was switched to oral clindamycin, which was continued for a total duration of six weeks (Table 1).

**Table 1:** Antibiotic sensitivity pattern of Streptococcus intermedius (member of the Streptococcus anginosus group) S: Susceptible

Antibiotic	Susceptibility
Penicillin	S
Amoxicillin	S
Amoxicillin / clavulanic acid	S
Ceftriaxone	S
Vancomycin	S
Doxycycline	S
Clindamycin	S

The patient showed good clinical recovery. Due to physeal involvement, long-term follow-up was arranged to monitor for potential growth disturbances.

## Discussion

Our case illustrates several important clinical lessons.

First, septic bursitis is uncommon in children and is mainly described in isolated case reports, with no available prevalence data. In contrast, it is more frequently reported in adult men, often associated with occupational kneeling or repetitive trauma [7,8,10]. Osteomyelitis, however, is more commonly encountered in the pediatric population [1]. Although superficial swelling may suggest bursitis, the epidemiological likelihood of osteomyelitis should remain an important consideration when evaluating atraumatic knee swelling in children.

Second, Brodie’s abscess represents a subacute, localized form of osteomyelitis characterized by an insidious onset and limited systemic inflammatory response. van der Naald et al., in a systematic review of 407 reported cases, found a median age at presentation of 17 years, with a male predominance (male: female ratio 2.1:1) and a median symptom duration of approximately 12 weeks prior to diagnosis. Pain was reported in 98% of cases and swelling in 53%, while 84% of patients were afebrile and fewer than 50% demonstrated elevated inflammatory markers [1]. This relative absence of systemic signs may result from the contained intraosseous nature of the infection, surrounded by a sclerotic bone reaction, thereby limiting systemic inflammatory response. Our patient presented with a subtle clinical picture and transient improvement under empiric antibiotic therapy, which may lead to false reassurance and delayed diagnosis.

Third, imaging plays a crucial role in the diagnostic process. Ultrasound may be helpful in identifying superficial fluid collections but cannot reliably exclude underlying bone infection. While MRI remains the gold standard for detecting intraosseous abscesses, assessing physeal involvement, and differentiating infection from neoplastic or inflammatory processes, conventional radiographs remain an important and accessible first-line diagnostic tool [10-12]. Despite understandable concern regarding radiation exposure in children, reluctance to obtain plain radiographs should not impede adequate diagnostic work-up. Contemporary dosimetry studies demonstrate that radiation exposure from pediatric extremity radiography is extremely low. Earl et al. reported effective doses of <0.001 mSv for distal extremity radiographs, corresponding to a negligible estimated lifetime cancer risk (approximately 1 in >10 million). This exposure is substantially lower than the average daily natural background radiation [13].

Finally, physeal extension underscores the importance of early recognition in pediatric patients, as growth disturbances may occur. Although overall outcomes are generally favorable following surgical debridement combined with antibiotic therapy, recurrence has been reported in approximately 15% of cases, necessitating long-term clinical and radiological follow-up [1].

Our case demonstrates that a plausible superficial diagnosis combined with minimal systemic features may create false reassurance. Persistent, recurrent, or atypical pain warrants reconsideration of the initial diagnosis and should prompt further imaging to exclude underlying osteomyelitis such as Brodie’s abscess.

## Conclusions

Brodie’s abscess should remain an important consideration in the differential diagnosis of children presenting with localized persistent or recurrent bone pain in the absence of trauma, even when fever or marked laboratory abnormalities are absent.

Early imaging is essential to avoid delayed diagnosis and potential complications. In particular, MRI plays a key role due to its high sensitivity for detecting intraosseous infection and associated soft-tissue involvement.
